# Alteration in Cellular Immunity after Chronic Hepatitis B Deteriorated into Severe Hepatitis and Its Significance

**DOI:** 10.5812/kowsar.1735-143x.760

**Published:** 2011-10-01

**Authors:** Gu Xibing, Yang Xiaojuan, Lu Zhonghua, Wang Juanhua

**Affiliations:** 1 Wuxi Hospital for Infectious Diseases, Wuxi, China

**Keywords:** Hepatitis B, Chronic, T-Lymphocytes, Killer Cells, Natural

## Abstract

**Background:**

It is difficult to predict what type of chronic hepatitis B (CHB) progresses to chronic severe hepatitis B.

**Objectives:**

This study aimed to observe changes in the HBV-specific and -nonspecific cellmediated immune responses after CHB deteriorates into severe hepatic disease and explore the significance of such changes.

**Patients and Methods:**

This study aimed to observe changes in the HBV-specific and -nonspecific cell-mediated immune responses after CHB deteriorates into severe hepatic disease and explore the significance of such changes.

**Results:**

In 49 of 255 CHB patients (19.22%), the disease developed into chronic severe hepatitis (early stage) an average of 10.06 ± 1.73 days after admission. CD4+ and NK cells levels in Group A were lower after progression into severe hepatitis than on the second day of admission (baseline) (P < 0.01). CD8+ cells and nonspecific CTL levels in Group A were higher after progression than at baseline (P < 0.01), and latter was higher than in Group B at baseline (P < 0.01); the levels of CD8+ cells and nonspecific CTLs in Group A after progression were significantly higher than those of Group B 10 days after admission (P < 0.01). There were no significant differences in HBV-specific CTL levels in Group A before and after progression to severe hepatitis (P > 0.05).

**Conclusions:**

Our results suggest that the immunological pathogenesis of chronic severe hepatitis B is related to significant rises in CD8+ and nonspecific CTL levels and that such increases predict that the disease will deteriorate into severe hepatitis.

## 1. Background

Clinically, liver disease can progress into chronic severe hepatitis B in some patients with CHB, even after admission and after receiving treatment. It is difficult to predict which CHB patients will develop chronic severe hepatitis.

## 2. Objectives

The pathogenesis of hepatitis B is associated with cellular immune responses that aim to remove HBV from the body [1]. If the resulting cellular immunity is too intensive, it can result in the necrosis of many liver cells and severe hepatitis. However, it is unknown whether HBV-specific or nonspecific cellular immune responses immediate the deterioration of CHB.

## 3. Patients and Methods

### 3.1. Subjects

We enrolled 255 CHB patients into this study from Jan. 2006 to Aug. 2010. This study was conducted in our hospital in Wuxi, China. Diagnoses of CHB and chronic severe hepatitis were made per the diagnostic criteria in the Program for Prevention and Treatment of Viral Hepatitis [2]. The requirements for enrollment included positivity for HBV DNA (HBV DNA > 103 copies/mL), alanine aminotransferase (ALT) > 2× upper limit of normal value, and age over 18 years. Exclusion criteria included patients with hepatitis A, C, D, or E infection; a medical history of autoimmune disease; alcohol abuse; medication with hepatotoxic agents; and use of antiviral agents, such as nucleoside analogs and interferons, and immune-enhancing or immunosuppressive agents, such as glucocorticoids.

The 255 CHB patients were divided into two groups. Group A consisted of 49 cases whose disease progressed to chronic severe hepatitis B; 35 were male (71.45%), the average age was 35.68 ± 9.63 years, and the average course of illness was 4.27 ± 2.40 years. Group B contained 206 cases in whom the disease did not progress to chronic severe hepatitis B; 147 were male (71.36%), the average age was 35.49 ± 10.09 years, and the average course of illness was 4.23 ± 2.32 years. There was no significant difference in sex, age, or course of illness between the groups (P > 0.05). There was no significant difference in ALT, HBV DNA, or HbeAg-positive rate between groups (P >0.05) on the second day of admission (Table 1). The two groups were comparable in every aspect. Thirty healthy blood donors were enrolled as controls; 22 were male (73.33%), and the average age was 35.18 ± 7.01 years.

**Table 1:** Comparison of ALT, Total Bilirubin (TBIL), Albumin (ALB), HBV DNA and HBeAg Positive Rate between Group A and Group B at Baseline (mean ± SD)

	**ALT a, u/L**	**TBIL a, μmol/L**	**ALB a, g/L**	**HBVDNA a, log10 Copies/mL**	**HBeAg a Positive, No. (%)**
Group A, (n = 49)	426.35 ± 182.31	38.25 ± 22.17	41.95 ± 3.31	5.58 ± 0.76	29 (59.18)
Group B, (n = 206)	423.61 ± 190.91	37.88 ± 21.01	42.03 ± 1.42	5.59 ± 0.81	132 (64.08)
t value	0.115	0.146	0.273	0.113	χ2 = 0.409
P value	< 0.05	< 0.05	< 0.05	< 0.05	< 0.05

^a^ aAbbreviations: ALB, albumin; ALT, alanine aminotransferase; HBeAg; hepatitis B e antigen ; HBVDNA, hepatitis B virus DNA; TBIL, total bilirubin

### 3.2. Treatment Methods

Both groups were treated with 200-mg silybin meglumine tablets (Jiangsu Zhongxing Pharmaceutical) orally three times daily to protect the liver. For patients whose disease deteriorated, antiviral treatments were given after the laboratory tests when indicated. For those whose disease did not progress into severe hepatitis by Day 20 after admission, antiviral therapy was given when the therapy was indicated.

### 3.3. Prothrombin Time (Activity)

Prothrombin time was measured by magnetic bead method (reagents and ACL-200 were purchased from Coulter, USA).

### 3.4. HBV Markers and HBV DNA

HBV markers (HBsAg, anti-HBs, HBeAg, anti-HBe, and anti-HBc) were measured by 1235 time-resolved fluorescence method; the reagents were purchased from Xinbo, Shanghai. HBV DNA was assayed by real-time fluorescence quantitative PCR; the reagents were purchased from Kehua, Shanghai.

### 3.5. Identification of HLA-A2 Allotypes

Fresh blood samples (100 μL) that were treated with heparin sodium were loaded into test and control tubes. After adding 10 μL HLA-A2-PE and the same type control, the tubes were incubated at room temperature for 30 min in the dark and analyzed on a Beckman-Coulter 3XL after hemolysis. Reagents were purchased from Proimmune, UK.

### 3.6. HBV-Specific CTL Test

**Figure 1:**
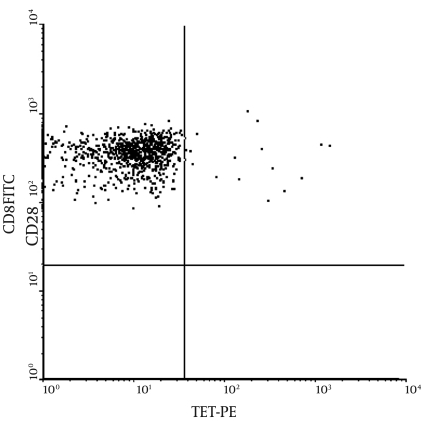
Flow Cytometry of HBV Specific CTL

HBV-specific CD8+ T cells were measured by HLA-peptide tetramer flow cytometry, the principle of which is as follows (3): The tetramer is labeled with phycoerythin (PE), and CD8+ T cells from peripheral blood that are specific for an epitope identify and bind short peptides in the antigen channel through the T cell receptor (TCR); these CD8+ T cells are detected by FITC-labeled anti-CD8, and HBV antigen-specific CD8+ T cells are detected by dual-color flow cytometry. (Figure 1)

Flow cytometry was performed as follows: 10 μL PElabeled HLA-peptide tetramer, anti-CD8-FITC, CD3-PC5, and 1 μL HBV core 18-27 antigenic peptide were loaded into test tubes; 100 μL heparin anti-coagulated blood was added and mixed well, and the tubes were incubated at room temperature for 20 min in the dark. After hemolysis and rinse steps, the samples were analyzed on a flow cytometer. A parallel control (without antibody to the antigenic peptide) was also run. Five thousand CD8+ cells were counted, gating on CD3+ lymphocytes, and CD8+ and HLA-peptide tetramer double-positive cells were counted as HBV-specific CD8+ cells, expressed as the percentage of total CD8+ cells. All reagents were purchased from Beckman Coulter.

### 3.7. T Cell Subgroup, Nonspecific CTLs, and NK Cells

**Figure 2:**
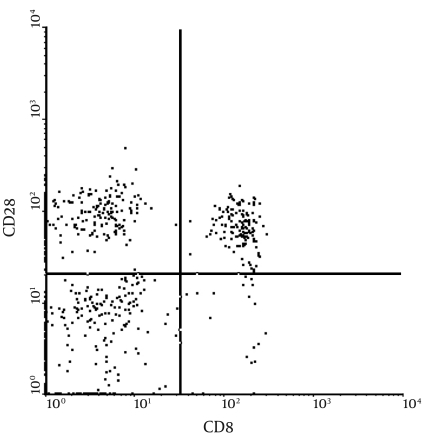
Flow Cytometry of Nonspecific CTL

EDTA-K2 anticoagulated fresh blood (100 μl) was loaded into test and control tubes, and 10 μL of monoclonal antibody (anti-CD3, anti-CD4, anti-CD8, anti-CD28, and anti-CD16 + 56; Beckman Coulter) and parallel nonspecific controls were added, respectively. The tubes were incubated at room temperature for 15 min in the dark and analyzed by flow cytometry after hemolysis. (Figure 2)

### 3.8. Statistical Analysis

SPSS 12.0 was used for the statistical analysis. Chi-square test was applied for categorical data, and t-test was used for numerical data, which were expressed as mean ± standard deviation (SD). The differences were considered significant when P < 0.05.

## 4. Results

### 4.1. Deterioration of CHB into Chronic Severe Hepatitis B

In 49 of the 255 CHB patients (19.22%), the disease progressed into early-stage chronic severe hepatitis B (Group A) an average of 10.06 ± 1.73 days after admission, with such symptoms as lassitude, decreased appetite, nausea, and vomiting; the mean TBIL was 338.51 ± 59.31 μmol/L, and prothrombin activity was 35.25 ± 2.91%. In the remaining 206 cases (80.78%), the disease did not progress into severe hepatitis B (Group B).

### 4.2. Comparison of T Cell Subgroups, Nonspecific CTLs, and NK Cells between CHB Patients and Controls

The T cell subgroup, nonspecific CTL, and NK cell levels on Day 2 of admission (baseline) in the 255 CHB patients and 30 healthy controls are listed in Table 2. The mean CD3+, CD4+, CD4+/CD8+, and NK cell levels in the patients with CHB were significantly lower versus the control group (P < 0.01), and the mean CD8 and nonspecific CTL levels in CHB patients were significantly higher compared with controls (P < 0.01). Table 3 shows the comparison of T cell subgroups, nonspecific CTLs, and NK cells at baseline between Groups A and B. The mean CD3+, CD4+, CD4+/CD8+, and NK cell levels in Group A were significantly lower than in the healthy control group (P < 0.01), and the mean CD8+ and nonspecific CTL levels in CHB patients were higher than in the healthy control group (P < 0.01).

The mean CD3+, CD4+, CD4+/CD8+, and NK cell counts in Group B were also significantly lower than in the control group (P < 0.01), and mean CD8+ and nonspecific CTL levels in Group B were higher than in the healthy control group (P < 0.01).

**Table 2:** Comparison of T Cell Subgroup, Non-Specific CTL a and NK a  Cells between 255 CHB Patients and Normal Controls (mean ± SD) at Baseline

	**CTLC**	**CD4^+^**	**CD8^+^**	**CD4^+^/CD8^+^**	**Non-Specific CTL**	**NK Cells**
CHB, (n = 255)	65.78 ± 2.76	33.51 ± .23	27.58 ± 90	1.23 ± 0.11	17.79 ± 1.80	12.55 ± .87
Healthy controls, (n = 30)	68.83 ± 5.51	37.21 ± 6.09	25.13 ± 4.78	1.54 ± 0.43	15.83 ± 4.99	15.04 ± 4.17
*t*value	4.991	6.552	5.131	8.679	4.261	5.818
*P*value	< 0.01	< 0.01	< 0.01	< 0.01	< 0.01	< 0.01

^a^ Abbreviation: CTL, cytotoxic T lymphocyte; NK, natural killer

**Table 3:** Comparison of Baseline T Cell Subgroup, Non-Specific CTL a and NK a Cells between Group A and Group B with Normal Controls (mean ± SD)

	**CD3^+^**	**CD4^+^**	**CD8^+^**	**CD4^+^/CD8^+^**	**Non-Specific CTL**	**NK Cells**
**Group A, (n = 49)**						
Value, mean ± SD	65.01 ± 1.27	30.61 ± 1.67	30.09 ± 1.21	1.02 ± 0.05	20.24 ± 1.57	10.33 ± 1.93
Healthy controls, mean ± SD (n = 30)	68.83 ± 5.51	37.21 ± 6.09	25.13 ± 4.78	1.54 ± 0.43	15.83 ± 4.99	15.04 ± 4.17
*t* value	4.315	6.591	6.351	7.662	5.207	6.816
*P* value	< 0.01	< 0.01	< 0.01	< 0.01	< 0.01	< 0.01
**Group B, (n = 206)**						
Value, mean ± SD	65.83 ± 3.07	34.23 ± 1.71	26.95 ± 1.51	1.28 ± 0.06	17.18 ± 1.28	13.08 ± 1.41
Healthy controls, mean ± SD (n = 30)	68.83 ± 5.51	37.21 ± 6.09	25.13 ± 4.78	1.54 ± 0.43	15.83 ± 4.99	15.04 ± 4.17
*t* value	4.255	5.311	3.997	7.367	2.989	5.078
*P*value	< 0.01	< 0.01	< 0.01	< 0.01	< 0.01	< 0.01

^a^ Abbreviation: CTL, cytotoxic T lymphocyte; NK, natural killer

### 4.3. Comparison of T Cell Subgroups, HBV-Specific CTL, Nonspecific CTLs, and NK Cells between Groups A and B

Of the 49 cases in Group A, 25 were positive for HLA-A2 (51.02%); of the 206 cases of Group B, 103 were positive for HLA-A2 (50%). HBV-specific CTLs were measured only in HLA-A2-positive patients. T cell subgroup, HBV-specific CTL, nonspecific CTL, and NK cell levels at baseline in both CHB groups are listed in Table 4. The mean levels of CD4+, CD4+/CD8+ and NK cells in Group A were significantly lower than in Group B (P < 0.01), and the mean CD8+ and nonspecific CTL levels in Group A were higher than in Group B (P < 0.01). Interestingly, there was no significant difference in HBV-specific CTL response between the two groups of patients with CHB i.e. the HBV-specific CTL response did not increase in the 49 patients before their disease deteriorated (P > 0.05).

The nonspecific and HBV-specific cellular immunity parameters, determined on an average of 10 days after admission-i.e. after the disease in 49 cases progressed to severe liver disease-are listed in Table 5. The mean CD4+, CD4+/CD8+, and NK cell levels in Group A were significantly lower than in Group B (P < 0.01), and the mean CD8+ and nonspecific CTL values in Group A were significantly higher than in Group B (P < 0.01). However, the HBV-specific CTL count was higher in Group A than in Group B (0.33 vs. 0.30) (P < 0.01).

**Table 4:** Comparison of Baseline T Cell Subgroup, HBV Specific CTL a Non-Specific CTL and NK a Cells between Group A and Group B (mean ± SD)

	**CD3^+^**	**CD4^+^**	**CD8^+^**	**CD4+/CD8^+^**	**Non-specific CTL**	**HBV specific CTL**	**NK cells**
Group A, (n = 49)	65.01 ± 1.27	30.61 ± 1.67	30.09 ± 1.21	1.02 ± 0.05	20.24 ± 1.57	0.32 ± 0.02(n = 25)	10.33 ± 1.93
Group B, (n = 206)	65.83 ± 3.07	34.23 ± 1.71	26.95 ± 1.51	1.28 ± 0.06	17.18 ± 1.28	0.31 ± 0.03(n = 103)	13.08 ± 1.41
t value	1.678	12.761	12.311	19.997	12.971	0.881	11.364
P value	> 0.05	< 0.01	< 0.01	< 0.01	< 0.01	> 0.05	< 0.01

^a^ Abbreviation: CTL, cytotoxic T lymphocyte; NK, natural killer

**Table 5:** Comparison of T Cell Subgroup, HBV Specific CTL a ,Non-Specific CTL and NK a Cells between Group A after the Disease Progressed to Chronic Severe Hepatitis B and Group B 10 Days after Admission (mean ± SD)

	**CD3^+^**	**CD4^+^**	**CD8^+^**	**CD4+/CD8^+^**	**Non-Specific CTL**	**HBV Specific CTL**	**NK Cells**
Group A, (n = 49)	65.42 ± 2.13	28.25 ± 1.66	33.38 ± 1.51	0.85 ± 0.06	23.47 ± 1.77	0.33 ± 0.03(n = 25)	7.02 ± 1.45
Group B, (n = 206)	65.87 ± 3.15	34.17 ± 1.72	26.97 ± 1.44	1.27 ± 0.08	17.21 ± 1.55	0.30 ± 0.03(n = 103)	13.07 ± 1.98
t value	0.898	19.515	24.765	28.561	21.956	3.588	20.10
P value	> 0.05	< 0.01	< 0.01	< 0.01	< 0.01	< 0.01	< 0.01

^a^ Abbreviation: CTL, cytotoxic T lymphocyte; NK, natural killer

### 4.4. Comparison of T Cell Subgroups, HBV Specific CTLs, Nonspecific CTLs, and NK Cells in Group A before and after Disease Progression to Severe Liver Disease

The T cell subgroup, HBV-specific CTL, nonspecific CTL, and NK cell levels in Group A before and after progression to chronic severe hepatitis B and Group B at baseline and 10 days after admission are listed in Table 6. After deterioration of the disease, the mean values of CD4+, CD4+/CD8+, and NK cell levels decreased significantly (P < 0.01), the change in CD3+ T cells was not significant (P > 0.05), and the mean values of CD8+ and nonspecific CTL increased significantly (P < 0.01), whereas the HBV-specific CTL response did not show any significant change (P > 0.05). All 7 parameters of cellular immunity in Group B did not show any significant changes between baseline and 10 days after admission (P > 0.05), except for a decrease in HBV-specific CTL responses (Table 6).

**Table 6:** Comparison of T Cell Subgroup, HBV Specific CTL a, Non-Specific CTL and NK a Cells in Group A after the Disease Progressed to Chronic Severe Hepatitis B, Group B 10 Days after Admission and the baseline of both Groups (mean ± SD)

	**CD3^+^**	**CD4^+^**	**CD8^+^**	**CD4^+^/CD8^+^**	**Non-Specific CTL**	**HBV Specific CTL**	**NK Cells**
**Group A, (n = 49)**							
Baseline	65.01 ± 1.27	30.61 ± 1.67	30.09 ± 1.21	1.02 ± 0.05	20.24 ± 1.57	0.32 ± 0.02 (n = 25)	10.33 ± 1.93
After progress	65.42 ± 2.13	28.25 ± 1.66	33.38 ± 1.51	0.85 ± 0.06	23.47 ± 1.77	0.33 ± 0.03 (n = 25)	7.02 ± 1.45
*t* value	1.125	6.441	10.887	11.567	8.83	0.971	9.594
*P* value	< 0.05	< 0.01	< 0.01	< 0.01	< 0.01	< 0.05	< 0.01
**Group B, (n = 206)**							
Baseline	65.83 ± 3.07	34.23 ± 1.71	26.95 ± 1.51	1.28 ± 0.06	17.18 ± 1.28	0.31 ± 0.03 (n=103)	13.08 ± 1.41
10 d after admission	65.87 ± 3.15	34.17 ± 1.72	26.97 ± 1.44	1.27 ± 0.08	17.21 ± 1.55	0.30 ± 0.03 (n=103)	13.07 ± 1.98
*t* value	0.161	0.492	0.251	0.664	0.283	2.86	0.059
*P* value	< 0.05	< 0.05	< 0.05	< 0.05	< 0.05	< 0.01	< 0.05

^a^ Abbreviation: CTL, cytotoxic T lymphocyte; NK, natural killer

## 5. Discussion

The results of the present study show that there were significant increases in peripheral blood CD8+ cells and nonspecific CTLs in patients with chronic severe hepatitis B (early stage), suggesting that the immunological pathogenesis of chronic severe hepatitis B is related to significant increases in CD8+ and nonspecific CTL levels and these increases can predict that CHB will progress to severe hepatitis. In China, most cases of severe chronic hepatitis result from HBV infection. Chronic severe hepatitis B progresses rapidly and is difficult to manage. The mortality rate of severe chronic hepatitis is over 50% [4][5], and 36.9% of cases with chronic severe hepatitis occurred on the basis of CHB [6]. Preventing or halting the deterioration can obviously improve the prognosis of CHB patients. To do so, it is necessary to determine or predict in which CHB patients the disease will progress to severe hepatitis so that corresponding measures can be taken. It is commonly believed that the pathogenesis of hepatitis B is the consequence of cellular immune responses that cause damage to the liver, although they are aimed primarily at removing HBV from the liver tissue [1]. The exacerbation of chronic hepatitis is mainly due to the action of CD8+ CTLs, which kill liver cells directly through double identification of major histocompatibility complex (MHC) antigen and hepatitis B antigen [7].

In an animal model of severe hepatitis, Ando et al. demonstrated that CD8+ cells cause the necrosis of many liver cells through cellular immunity [8]. However, recent studies suggest that cellular immune mechanisms for eliminating HBV can be divided into specific and nonspecific responses [3]. Specific cellular immune responses of the body that are induced by the virus may be an important factor in eliminating the virus [9]. There are two routes for virus-specific CTLs to remove HBV: cytotoxic and noncytolytic [10]. In the noncytotoxic route, specific T lymphocytes only clear the virus from target cells, without injuring the target cell itself. In the process of clearing the virus by specific T cells, the noncytolytic route may play a more important role [11]. Nonspecific cellular immunity is relatively weak in clearing HBV, but it may cause liver cell injuries in the process [12][13][14]. Thus, we studied the changes in HBV-specific and nonspecific cellular immune responses before and after CHB progressed to chronic severe hepatitis B to provide evidence for the rational treatment, control, and prevention of deterioration of CHB.

In this study, CD3+, CD4+, CD4+:CD8+ ratio, and NK cell levels in 255 CHB patients were lower than in healthy controls (P < 0.01). CD8+ and nonspecific CTL levels were higher than in healthy controls (P < 0.01), which clearly indicates that cellular immunity in CHB patients is dysfunctional. The levels of CD4+ and NK cells of Group A (i.e., patients whose disease progressed to severe hepatitis) were lower than in the same patients on Day 2 (baseline) of admission (P < 0.01), which were lower than Group B baseline levels (P < 0.01), and the latter was lower than in the healthy control group (P < 0.01). These levels in Group A after progression to severe hepatitis were lower than in Group B 10 days after admission (P < 0.01). The levels of CD8+ and nonspecific CTLs in Group A after progression to severe hepatitis were higher than in the same group at baseline (P < 0.01), which were higher than the baseline levels in Group B (P < 0.01), and the latter was higher than in the healthy control group (P < 0.01). These parameters in Group A after deterioration to severe hepatitis were higher than in Group B 10 days after admission (P < 0.01).

In patients with chronic severe hepatitis B, CD4+ levels decrease and CD8+ levels increase [15]. The CD8+ T cells that increase in patients with viral hepatitis are mainly CTL [15]. Our study also demonstrates this phenomenon. HBV-specific CTL levels were not associated with deterioration of the disease, since there was no significant difference in specific CTL levels between Groups A and B at baseline (P > 0.05) or in Group A before and after the disease progressed to severe hepatitis (P > 0.05). On the other hand, in patients with severe chronic hepatitis, NK cells may migrate to the target cells via chemotaxis and exert antiviral actions or kill liver cells directly, which can reduce NK cell numbers [16]. This study shows that peripheral blood CD8+ and nonspecific CTLs in patients with chronic severe hepatitis B (early stage) increased significantly compared with CHB patients whose disease did not deteriorate, whereas HBV-specific CTLs did not change, which suggests that the immunological pathogenesis of chronic severe hepatitis B is associated with a significant increase in CD8+ and nonspecific CTLs. In addition, before the disease in Group A patients progressed to severe hepatitis, CD8+ and nonspecific CTL levels had already increased markedly. Therefore, significant increases in CD8+ and nonspecific CTL levels in CHB patients may predict deterioration of CHB to severe hepatitis. When the deterioration occurs, the symptoms of patients worsen. Glucocorticoids should be considered in the treatment of such patients to decrease the excessively high levels of CD8+ T cells and nonspecific CTLs [17] and prevent CHB patients from developing chronic severe hepatitis.

The weaknesses of this study are that we did not test cellular immunity in the liver tissue of patients, the follow-up period was too short, and the sample size was not large enough. Larger-scale, better-designed studies are needed for further exploration of the mechanisms of deterioration of chronic hepatitis B. In conclusion, this study shows that the deterioration of CHB may be associated with significantly increased levels of CD8+ T cells and nonspecific CTLs, which may predict deterioration of CHB.
